# Effect of the addition of yacon flour (*Smallanthus sonchifolius*) and freezing on baking performance

**DOI:** 10.1002/jsfa.70846

**Published:** 2026-06-29

**Authors:** Gabriel A. Atanazio‐Silva, Raquel Garzon, Giulliana P. Torrano, Carmen C. Tadini, Cristina M. Rosell

**Affiliations:** ^1^ Escola Politécnica, Department of Chemical Engineering Universidade de São Paulo São Paulo Brazil; ^2^ Institute of Agrochemistry and Food Technology (IATA‐CSIC) Paterna Spain; ^3^ Food Research Center (FoRC) Universidade de São Paulo São Paulo Brazil; ^4^ Department of Food and Human Nutritional Sciences University of Manitoba Winnipeg Canada; ^5^ Richardson Center for Food Technology and Research University of Manitoba Winnipeg Canada

**Keywords:** yacon, cryoprotectant, freezing, fructans

## Abstract

**BACKGROUND:**

Yacon (*Smallanthus sonchifolius*), a tuber rich in fructans, presents potential for application in breadmaking due to its nutritional properties and cryoprotectant activity. The aim of this study was to evaluate the incorporation of yacon flour in bread formulations (50 g/kg and 100 g/kg on flour mixture basis) and its impact on baking processes involving dough freezing.

**RESULTS:**

Dough consistency showed that yacon inclusion decreased the water needed to achieve a proper consistency; however, this incorporation also weakened the gluten network structure and delayed starch gelatinization. Low yacon replacement enhanced fermentation kinetics and preserved dough volume increase after the freezing process. Meanwhile, moderate yacon substitution had a negative influence on dough volume increase after freezing, a behavior attributed to a weakened gluten network. The analysis of thermal properties revealed a higher gelatinization temperature peak of starch in doughs containing yacon. Furthermore, most of the gelatinization, freezing, and thawing enthalpies were lower for doughs containing yacon due to reduced freezable water content and water‐holding capacity of this flour. The bread characterization evidenced that the yacon addition darkened the bread's color, increased its hardness and reduced its springiness, while the process type mainly affects structural and dimensional attributes.

**CONCLUSIONS:**

In summary, the results suggest that yacon flour can be incorporated into breadmaking to provide nutritional benefits and enhance the resistance of the dough to freezing processes. However, low concentrations of yacon flour are recommended, around 50 g/kg of wheat flour substitution, to ensure that the breadmaking and final properties of the bread will not be compromised. © 2026 The Author(s). *Journal of the Science of Food and Agriculture* published by John Wiley & Sons Ltd on behalf of Society of Chemical Industry.

## INTRODUCTION

Yacon (*Smallanthus sonchifolius*), a tuber native to the Andean region, has garnered considerable attention as a functional food due to its unique composition.^1^ Yacon is mainly used as flour because of the short shelf life of the fresh tuber due to its high moisture content (830–900 g/kg wet basis).^2^, ^3^ Yacon is rich in carbohydrates (900 g/kg dry basis), primarily fructooligosaccharides (FOSs) and inulin, both fructan‐type carbohydrates that exhibit prebiotic properties and immunomodulatory activities, justifying their use in dietary applications.^1^, ^4^, ^5^ The ratio between these fructans changes depending on growth and storage conditions.^3^

Several studies have explored the incorporation of yacon flour into bakery products, demonstrating enhancements in its nutritional value. For instance, Rolim *et al*.^6^ reported that the use of yacon flour to enrich wheat bread, with yacon flour incorporation of 60 or 110 g/kg, on wheat flour basis, leads to bread with higher fiber content and reduced glycemic index when compared to the control sample. The first effect occurs because of the high fiber content naturally contained in this flour, while the second is possibly associated with fructans' gelatinization during baking, hindering the enzymatic activity of amylose, reducing the conversion of these carbohydrates into glucose molecules. Moreover, yacon flour inclusion enhanced the prebiotic nature of bread, as evidenced by the growth of probiotic bacteria, especially *Lactobacillus* species. Derkanosova *et al*.^7^ studied the addition of three different concentrations of yacon flour (35, 70 and 105 g/kg, on wheat flour basis) in wheat bread formulations; as a result, the enriched breads exhibited an enhanced yeast activity mainly due to fructose, phosphorus, and magnesium present in yacon. However, they also found that higher levels of yacon flour in dough can darken both crumb and crust, as well as reduce the crumb's porosity and specific volume of the final product. Therefore, despite nutritional and technological benefits provided by yacon flour, the authors recommend an adequate incorporation range of 35–70 g/kg to avoid negative effects on the organoleptic and physicochemical properties of bread.

Viell *et al*.^8^ evaluated sensory attributes of gluten‐free breads produced with a gluten‐free mix (GFM), composed of rice flour (520 g/kg), potato starch (360 g/kg) and cassava starch (120 g/kg), and enriched with yacon and teff flour. At a yacon flour substitution of 300 g/kg, on flour mixture basis, bread exhibited overall acceptance and sensory properties like the control sample. Nevertheless, a higher substitution of 600 g/kg led to lower overall acceptance, mainly due to texture, taste and color. Lancetti *et al*.^5^ assessed different drying conditions to obtain yacon flour and its effects in starch‐based gluten‐free muffins, with substitutions of 50 g/kg and 100 g/kg of corn starch by yacon flour, on starch‐flour mixture basis, and they concluded that its incorporation increased the cohesiveness and darkened the crumb of enriched muffins. Up to now, yacon flour applications have mainly relied on the nutritional benefits that this root can provide. However, yacon flour also possesses valuable technological properties, including excellent water‐holding and swelling capacities.^5^ For instance, Derkanosova *et al*.^7^ found that wheat breads enriched with an addition of 70 g/kg of yacon had 1.4 times less crumb friability than the control sample after 48 h of storage, indicating a longer freshness preservation after yacon addition.

Beyond these nutritional and structural effects, a notable technological potential use for yacon flour is its cryoprotective action during dough or bread freezing, since the application of inulin and FOS as effective cryoprotectants in frozen bakery systems has been widely reported.^9^, ^10^, ^11^ Considering the breadmaking process, dough freezing leads to ice crystal formation within the dough matrix, causing mechanical damage to the gluten network and yeast cells, resulting in reduced gas retention, smaller loaf volumes, and impaired crumb structure.^12^ These quality losses are largely associated with starch retrogradation and moisture migration, phenomena that are further exacerbated in partially baked breads, resulting in accelerated staling and loss of sensory attributes such as texture, flavor, and aroma during storage.^13^, ^14^ In this context, fructans act by reducing freezable water, limiting ice crystal growth, stabilizing cellular structures, and retarding starch retrogradation, thereby improving fermentation performance, structural integrity, and shelf‐life stability.^9^, ^10^, ^11^, ^12^, ^13^ Unlike isolated cryoprotective additives, yacon flour provides these technological functionalities concomitantly with dietary fiber enrichment and potential prebiotic effects, supporting its application as a multifunctional ingredient in frozen and partially baked bread systems.

Therefore, improving technological properties of doughs intended for freezing or partial baking is essential for maintaining product quality and extending shelf life. Despite high fructan content in yacon and its cryoprotective potential, there is a lack in the literature concerning the application of yacon flour in frozen bakery systems. Thus, considering the nutritional and technological benefits of yacon, this study aims to evaluate its potential role as a cryoprotectant in breadmaking, comprising freezing at both the dough and partially baked bread. This study aims to broaden the scientific knowledge regarding the cryoprotective potential of yacon flour in breadmaking through the combined evaluation of rheological, fermentative, thermal, and final bread quality properties. For this work, two levels of yacon flour substitution were tested (50 g/kg and 100 g/kg, on flour mixture basis), while the direct breadmaking without freezing was used as a reference.

## MATERIAL AND METHODS

### Material

For this study, wheat flour (La Meta, Lleida, Spain), commercial salt, baker's dry yeast (Lesaffre Ibérica S.A., Valladolid, Spain), tap water, and yacon flour were used. Yacon flour was prepared from roots purchased from a local market in São Paulo, Brazil, according to the procedure illustrated in Fig. 1. Briefly, the roots were washed, sanitized in a 200 ppm chlorine solution for 10 min,^6^ sliced, and treated with a 2 g/L citric acid solution for 3 min, to reduce enzymatic browning. The slices were dried using a LM‐ES20 drying oven (Labmaq, Ribeirão Preto, Brazil) at 60 °C and 20% relative humidity for 8 h, following Marques *et al*.^15^ The dried slices were milled in a MA600/CF mill (Marconi, Piracicaba, Brazil), and the resulting flour was sieved through a 1‐mm mesh sieve. Finally, the yacon flour was stored in vacuum‐sealed aluminum pouches.

**Figure 1:**
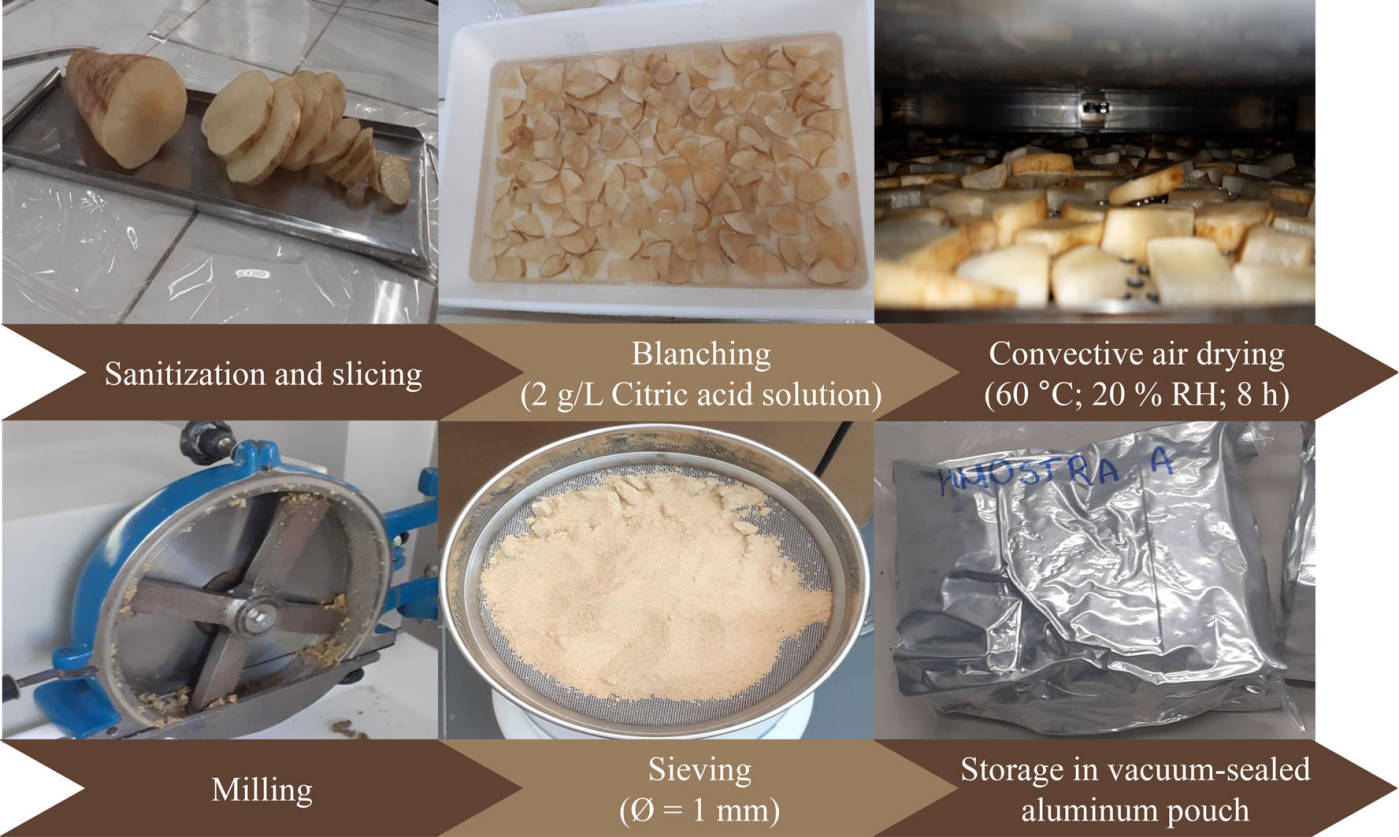
Diagram of yacon flour preparation procedure.

### Flour characterization

The moisture of yacon and wheat flour was determined, in triplicate, according to the standard method^16^ defined by the ICC (International Association for Cereal Science and Technology). Fructan content of yacon flour was quantified using the commercial Fructan HK assay kit (Megazyme, Wicklow, Ireland), in triplicate. For the interpretation of the results and further discussion, values for the other compounds of yacon flour were based on those reported in the literature.^6^, ^17^, ^18^, ^19^

The yacon and wheat flour particle size test was conducted as described in Collar *et al*.^20^ using the following sieves: 850 μm, 710 μm, 600 μm, 425 μm, 300 μm, 180 μm and 80 μm. For the test, 100 g of flour was placed in an electromagnetic sieve shaker IRIS FTL‐0200 (Filtra Vibración, Badalona, Spain) for 10 min. The mean particle diameter was calculated as the Sauter diameter by the analytical method of Kunii and Levenspiel^21^ and the particle size distribution was performed by the graphical method to identify the diameters below which 10% (*d*_10_), 50% (*d*_50_), and 90% (*d*_90_) of the particles have a smaller size.^22^

### Dough consistency

Mixolab (CHOPIN, Villeneuve‐la‐Garenne, France) was used to record the dough's rheological properties and determine the amount of water required to achieve the wheat dough's standard consistency of 1.1 N m (± 0.05 N m).^23^ The protocol ‘Chopin+’ was carried out, in duplicate, using 75 g of dough. This protocol involves a controlled mixing and heating process that quantifies water absorption capacity (WA), development time (*t*_1_), stability, time until peak *C*_Y_ (*t*_Y_), and gelatinization start time (*t*_3_), and extracts key torque parameters (*C*_1_, *C*_Y_, *C*_2_, *C*_3_, *C*_4_, and *C*_5_). These parameters collectively provide a comprehensive characterization of the dough's behavior during the various stages of mixing and thermal transitions, including initial mixing, protein weakening, starch gelatinization, enzymatic degradation, and starch retrogradation.

### Breadmaking

The basic recipe was based on 100 g of wheat flour, containing 10 g/kg dry yeast and 15 g/kg salt, and the water amount determined by the Mixolab analysis. For the recipes formulated with yacon flour, wheat flour was replaced by yacon flour (50 or 100 g/kg). All ingredients were mixed in a planetary mixer (N50; HOBART, Hillsboro, USA) at speed level 2. Dough was divided into 40 g pieces that were manually shaped to ensure uniformity and placed in molds with dimensions of 80 mm × 40 mm × 40 mm (Nordia, Pontcharra, France).

For the direct process, the molds were placed in a fermentation chamber (Salva Industrial, Gipuzkoa, Spain), at 30 °C and a relative moisture of 70%, for 45 min. Baking was done in an electric oven (F106; FM Industrial, Lucena, Spain), preheated at 200 °C, for 14 min. Finally, the loaves of bread were cooled at room temperature for 1 h.

For the freezing process, the dough was frozen in a vertical static freezer at −20 °C immediately after the shaping stage to avoid early fermentation. The samples were placed individually on the same tray, spaced far enough apart to allow uniform cold air exposure during freezing. Subsequently, the dough was thawed at room temperature for 70 min, and then fermented and baked, as previously described for the direct process.

To obtain the frozen partially baked bread, the direct process was followed for mixing and proofing. Baking was performed for 9.8 min, which corresponds to 70% of the total baking time applied in the direct process.^24^ Following part‐baking, the bread was cooled at room temperature for 40 min and frozen under the same conditions applied to frozen dough. After 7 days, the partially baked bread was thawed for 70 min at room temperature and then baked for 4.2 min.

Temperature was monitored throughout all breadmaking processes by thermocouples inserted into the geometric center of the samples, and data were recorded by a N2014 datalogger (COMARK, Massachusetts, USA) for comparison of cooling behavior among formulations. Two batches of breadmaking were performed for each recipe and process. Codes applied to samples were: W for wheat and Y for yacon, followed by 5 or 10 to indicate the replacement of 50 or 100 g/kg of wheat flour. To identify the freezing process, codes were followed by F for dough freezing and FP for frozen partially baked bread.

### Fermentation kinetics

To determine the necessary fermentation time, the fermentation kinetics of the dough were monitored as described in Garzon *et al*.,^25^ with modifications. In brief, two samples of 1 g of dough were placed into 10 mL graduated cylinders. The increase in dough volume at 30 °C and 70% relative humidity was recorded every 10 min for a total period of 70 min. The parameters defined included: the dough volume increase (∆*V*), corresponding to the ratio between dough volume at the end and beginning of fermentation, and the optimum fermentation time (OFT, in minutes), defined as the time required for the dough to reach 75% of its maximum volume.

### Thermal properties

To evaluate the influence of each process type and yacon content on the thermal behavior of the dough, a differential scanning calorimetry (DSC) technique was applied using an DSC Q2000 (TA Instruments, New Castle, DE, USA), in duplicate. Dough samples (10 mg) prepared without yeast were weighed into aluminum pans (Tzero Pan 901683.901; TA Instruments). Heating and cooling processes were defined to simulate the breadmaking process, using a heating and cooling rate of 5 °C/min. An empty pan was used as a reference for the test, which was kept the same for all the samples. For the direct process, the dough was heated from 30 °C to 120 °C. For the freezing process, the dough was cooled from 30 °C to −30 °C and then heated to 120 °C. Finally, for the part‐baking and freezing process, the dough was heated from 30 °C to 100 °C, then cooled to −30 °C and finally heated to 120 °C. Parameters defined included: Peak temperature and enthalpies corresponding to starch gelatinization (*T*_G_ and Δ*H*_G_), freezing (*T*_F_ and Δ*H*_F_), and thawing (*T*_T_ and Δ*H*_T_) of the dough. To enable a proper comparison, the gelatinization enthalpy was normalized using the starch content, while the freezing and thawing enthalpies were standardized based on the dry matter of each formulation. Moreover, the freezable water content in each sample was estimated using the thawing enthalpy (wet basis), the enthalpy of fusion of water, and the mass of sample inside of the pan.

### Bread characterization

Moisture, texture, color and crumb structure were assessed. The moisture content using both the bread crumb and crust was determined, in triplicate, according to the ICC standard method.^16^ The parameters of hardness, springiness, cohesiveness, chewiness and resilience were recorded on the TA.XT‐Plus Texture Analysis (Stable Micro Systems Ltd, Godalming, UK) equipped with a 5 kg load cell. Texture was performed in a 10 mm thickness central slice, after removing the crust. During the texture profile analysis (TPA) test, the center of the crumb was compressed twice with a 25 mm aluminum cylindrical probe at a speed of 1 mm/s and an interval of 30 s between compressions.^26^ The texture parameters were recorded using Texture Expert software and the average results of five replicates were calculated following Carr and Tadini.^27^

The crumb color was measured using the CIE‐L*a*b* color space using a Minolta colorimeter (Chromameter CR‐400/410; Konica Minolta, Tokyo, Japan) (D65 illumination and 10° viewing angle) after calibration using a white calibration plate (L* = 96.9, a* = −0.04, b* = 1.84). The color parameters indicate L* the lightness (100 = white and 0 = black), a* the tonality from green (−) to red (+) axis, and b* the tonality from blue (−) to yellow (+) axis. The measurements were performed on two slices of each loaf, and three loaves were selected from each batch, resulting in six replicates.^25^ To compare the different breads, the total color difference (Δ*E*) was calculated using the following equation

$$\Delta E=\sqrt{{\left(L_{r}^{*}-L_{s}^{*}\right)}^{2}+{\left(a_{r}^{*}-a_{s}^{*}\right)}^{2}+{\left(b_{r}^{*}-b_{s}^{*}\right)}^{2}}$$

where $L_{r}^{*}$, $a_{r}^{*}$, $b_{r}^{*}$ are color parameters of reference bread W, and $L_{s}^{*}$, $a_{s}^{*}$, $b_{s}^{*}$ are color parameters of the samples.^25^

The structure of the bread crumb was analyzed using an image analysis system (ImageJ; NIH, Bethesda, MD, USA), as previously described by Morreale *et al*.,^26^ in triplicate. The data acquired from the analysis of the crumb structure (two‐dimensional (2D) area of the slice) were applied to compare the different breads.

### Statistical analyses

The results obtained are reported as the mean and standard deviation of the measurements. The software Statgraphics Centurion XVII (Statgraphics Technologies, Inc., Virginia, USA) was employed to perform both the analysis of variance (ANOVA) and the multivariate analysis of variance (MANOVA) of the data, with a comparison between the means according to the yacon content and process type, applying Tukey's test with a 95% confidence level. Furthermore, the same software was used to conduct a principal component analysis (PCA) on the final bread characteristics (texture, color, and structure), using standardized data (mean‐centered and scaled to unit variance), and principal components (PCs) with eigenvalues greater than one were retained. Correlations among formulations, processing conditions, and bread properties were interpreted using a biplot of the two PCs identified.

## RESULTS AND DISCUSSION

### Flour characterization

The characteristics of wheat and yacon flours are shown in Table 1. The fructan content of yacon flour (456.5 g/kg) was consistent with values previously reported in the literature, ranging from 286 to 553 g/kg,^18^, ^19^, ^28^ suggesting that the methodology adopted for yacon flour production was adequate. The moisture content of wheat flour was higher than that of yacon flour. Differences in particle size were observed between the flours, with yacon flour exhibiting a larger distribution and mean particle size (175 μm) than wheat flour (106 μm). The larger particle size of yacon flour, combined with the hydrophilicity of fructans, may affect water redistribution and availability during mixing, thus altering flour hydration and potentially hindering gluten network development.^29^, ^30^

**Table 1:** Particle sizes (*d*_10_, *d*_50_, *d*_90_), average particle size ($\bar{d}$), fructan content (FC) and moisture of flour

Flour type	*d*_10_ (μm)	*d*_50_ (μm)	*d*_90_ (μm)	$\bar{d}$ (μm)	FC (g/kg dry basis)	Moisture (g/kg)
Wheat	80	124	168	106	—	136.0 ± 1.8
Yacon	100	243	634	175	456.5 ± 16.9	64.4 ± 0.8

### Dough consistency

Dough consistency was assessed using Mixolab, whose parameters are shown in Table 2. Compared to the control dough (W), the water absorption of Y5 and Y10 doughs decreased 7.3% and 16.4%, respectively, being directly associated with the increase in yacon content. Therefore, the water required to hydrate the flour and reorganize the gluten protein in the network structure probably decreased due to the reduction of wheat flour content and the plasticizing potential of yacon's fructans.^29^ This water reduction may also be related to the partial replacement of wheat flour with yacon flour, which does not form a gluten network, and to the hydrophilic nature of yacon, which may affect water binding.^30^ Although dough stability showed a numerical increase in Y10, no significant differences were detected among the samples (*P =* 0.054), suggesting that the incorporation of yacon flour did not markedly disrupt the stability over the mixing period.

**Table 2:** Parameters obtained from Mixolab of dough with partial replacement of wheat by yacon flour

Sample	W	Y5	Y10	*P*‐Value
WA (%)	55.45 ± 0.15^a^	51.42 ± 0.00^b^	46.34 ± 0.15^c^	**0.000**
*C*_1_ (N m)	1.12 ± 0.01	1.10 ± 0.02	1.06 ± 0.03	0.170
*t*_1_ (min)	4.86 ± 0.44	4.51 ± 0.27	4.82 ± 0.16	0.543
Stability (min)	8.66 ± 0.06	8.65 ± 0.52	9.76 ± 0.01	0.054
*C*_Y_ (N m)	—	1.15 ± 0.02	1.15 ± 0.00	1.000
*t*_Y_ (min)	—	7.12 ± 0.38	7.06 ± 0.53	0.273
*C*_2_ (N m)	0.41 ± 0.00^a^	0.30 ± 0.01^b^	0.27 ± 0.02^b^	**0.002**
*C*_3_ (N m)	2.00 ± 0.01	1.96 ± 0.04	1.90 ± 0.04	0.132
*t*_3_ (min)	26.08 ± 0.47^c^	27.29 ± 0.11^b^	28.12 ± 0.06^a^	**0.000**
*C*_4_ (N m)	1.92 ± 0.03	1.88 ± 0.03	—	0.302
*C*_5_ (N m)	2.10 ± 0.04	1.96 ± 0.03	1.98 ± 0.04	0.057
*α* (N m/min)	−0.08 ± 0.00^a^	−0.10 ± 0.00^b^	−0.09 ± 0.00^b^	**0.020**
*β* (N m/min)	0.37 ± 0.01^a^	0.32 ± 0.02^b^	0.33 ± 0.00^b^	**0.050**
*γ* (N m/min)	−0.01 ± 0.00	−0.02 ± 0.00	−0.02 ± 0.00	0.350

*Note*: Mean values within each row with the same letters are not significantly different (*P* > 0.05). Letters are included when statistically significant differences are observed. The values in bold correspond to those for which a significant difference was found (*p* ≤ 0.05).

Abbreviations: water absorption capacity (WA), development time (*t*_1_), stability, time until peak *C*_Y_ (*t*_Y_), gelatinization start time (*t*_3_), torque parameters (*C*_1_, *C*_*Y*_, *C*_2_, *C*_3_, *C*_4_ and *C*_5_) and slope parameters (*α*, *β*, and *γ*) of the dough produced with wheat flour only (W), with 50 g/kg (Y5) and with 100 g/kg (Y10) of yacon flour.

The incorporation of yacon promoted the occurrence of a second hydration peak (*C*_Y_) during the mixing, probably associated with the hydration of yacon flour, which increases its mobility and consequently facilitates the interaction between both flours. As a possible consequence, the fructans may compete for water and integrate into the gluten network through bonds with gluten proteins, in line with previous findings on the integration of inulin into wheat dough.^30^ Justifying the decreased resistance of the gluten network to heating and mechanical stress (evidenced by *C*_2_) after yacon incorporation.

Furthermore, the time necessary to reach the gelatinization peak (*t*_3_) was longer for doughs containing yacon, with increases of 4.6% and 7.8% for Y5 and Y10, respectively. Suggesting that the fructans surrounding the starch granules may inhibit the wheat starch gelatinization due to the competition for available water, thereby impairing the swelling of starch granules during heating. This delay was also reflected in the thermal analysis, with higher gelatinization temperature peaks, reinforcing the resistance to gelatinization of enriched doughs, as discussed later. Additionally, analysis of the slope parameters (*α*, *β*, and *γ*) revealed that *α* was significantly different in the control sample (W) compared to Y5 and Y10, indicating a more pronounced protein weakening rate in yacon‐containing formulations. Conversely, *β* and *γ* did not show significant differences (*p* > 0.05), suggesting that the starch pasting and final cooking stages were similarly affected in all doughs.

### Breadmaking process comprising a freezing stage

Figure 2 shows the temperature profiles monitored at the center of the dough during the freezing, thawing, and baking stages, allowing comparison of the cooling and heating behavior among formulations throughout breadmaking. Figure 2(A) (direct process) shows the steady increase in temperature from the fermentation stage (30 °C) to the final internal baking temperature (95 °C). This linear and uniform rise was consistent across the samples, suggesting similar thermal behaviors under continuous heat input in all formulations. In Fig. 2(B),(C), an initial cooling phase occurred until the dough reached subzero temperatures, followed by a period during which the temperature remained constant during the dough's frozen storage. Although the overall cooling curves looked similar, the recipes containing yacon (Y5F and Y10F) reached lower temperatures faster during cooling. A faster drop in temperature was observed in the Y10F dough, and a faster heating during the baking stage, possibly reflecting its lower water absorption capacity and hence altered heat transfer properties. In Fig. 2(D),(E), the dough was initially partially baked, then frozen, and subsequently underwent a second baking after thawing. Again, the presence of yacon induced faster freezing and faster increase of temperature during the second baking (Y5FP and Y10FP). This earlier temperature rise could reflect the additional sugars in yacon flour, which may enhance heat absorption and promote earlier crust formation, as well as the reduced water content of doughs containing yacon flour, which can increase thermal conductivity.^31^, ^32^ Allowing heat to penetrate the dough center at a faster rate compared to the wheat in the absence of yacon. The reduction in water availability in enriched doughs was further supported by DSC thermal analyses, which showed lower freezable water contents in samples containing yacon flour. This behavior may have contributed to the faster temperature reduction observed during freezing, since lower amounts of freezable water reduce the latent heat associated with ice crystallization.

**Figure 2:**
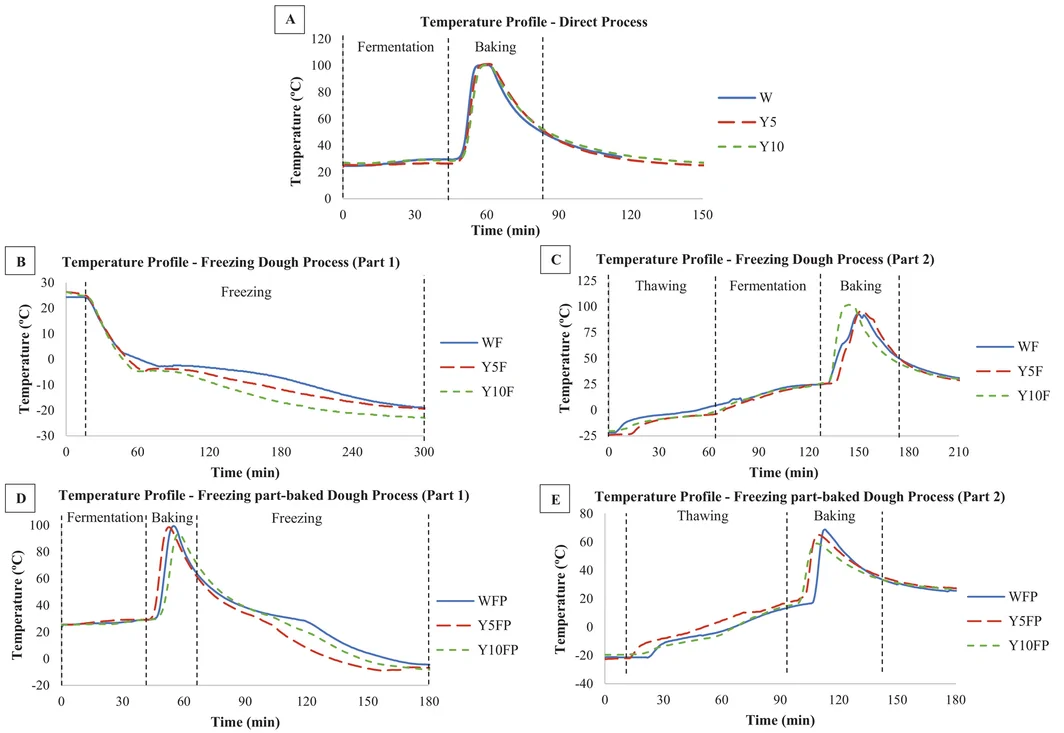
Temperature profiles recorded during the direct process (A); the freezing dough process part 1 (B) and part 2 (C); the freezing part‐baked process part 1 (D) and part 2 (E), of the dough produced with wheat flour (W), and containing 50 g/kg (Y5) or 100 g/kg (Y10) of yacon flour.

### Fermentation kinetics

To assess the effects of yacon flour incorporation and freezing on the fermentation kinetics, the dough volume was recorded (Table 3). Although the gluten network of the formulations containing yacon was weaker due to gluten dilution, the W and Y5 formulations presented equal fermentation performances, indicating that wheat replacement by 50 g/kg of yacon flour did not have a significant effect on the fermentation. Probably, part of the fructans were fermented by the yeast, improving the fermentation process and counterbalancing the weakened gluten network.^7^ Morreale *et al*.^32^ also reported that the fructans fermentation by yeast was dependent on their degree of polymerization. The dough with 100 g/kg of yacon flour (Y10) exhibited a slight decrease of 8% in dough volume increase compared to the control sample (W). Suggesting that the reduction in the gluten content, combined with the lower availability of water, due to the water‐binding capacity of fructans, may lead to gluten network weakening, negatively impacting dough viscoelasticity and gas retention during fermentation.

**Table 3:** Fermentation kinetics of wheat dough in the presence of different amounts of yacon flour, and the impact of the freezing process on the dough fermentation

Process type	Samples	Dough volume increase (%)	OFT (min)
Direct	W	150 ± 8^aA^	38.2 ± 2.3^aA^
	Y5	148 ± 5^abA^	38.2 ± 2.2^aA^
	Y10	138 ± 5^bA^	36.3 ± 0.7^aA^
Freezing the dough	WF	138 ± 2^aB^	30.1 ± 1.4^aB^
	Y5F	140 ± 0^aA^	31.1 ± 0.4^aB^
	Y10F	90 ± 0^bB^	23.4 ± 0.0^bB^
*P*‐Value	Process type	**0.000**	**0.000**
	Yacon flour	**0.000**	**0.000**

*Note*: Mean values within each process type with the same lowercase letter are not significantly different in relation to yacon flour content (*p* > 0.05). Mean values with the same capital letters are not significantly different in relation to process type (*p* > 0.05). The values in bold correspond to those for which a significant difference was found (*p* < 0.05).

Abbreviations: wheat (W), containing 50 g/kg yacon flour (Y5), containing 100 g/kg yacon flour (Y10), freezing the dough process (F), optimum fermentation time (OFT).

For the cases involving freezing, the results obtained were significantly different. Freezing impacted the fermentation ability of the wheat dough (WF), leading to a slight reduction of 8% in the dough expansion when compared with W. Regarding the inclusion of yacon flour, Y10F considerably affected the fermentation performance, resulting in a significant decrease of 40% in dough expansion compared to W. Likely, the formation of ice crystals during the freezing process magnified the impact of both dilution and weakening of gluten. Furthermore, the Y5F sample exhibited similar fermentation performance to Y5. This result reinforces the previous reports of inulin and FOS acting as cryoprotectants during the freezing process, protecting the yeast cells and preserving the dough growth.^9^, ^11^ However, for the Y10F sample, the cryoprotective activity was not effective in overcoming the weakness of the gluten network, resulting in a considerable reduction of 34.8% in dough expansion compared to Y10. Therefore, although fructans' content contributes to maintaining the fermentative activity of yeast cells, the damaged gluten network was unable to expand sufficiently and retain the gas produced, resulting in reduced dough growth.

### Thermal properties

The impact of fructans on starch gelatinization and water‐related thermal transitions was evaluated through DSC measurements (Table 4). The yacon presence significantly affected the peak temperatures and enthalpies of starch gelatinization (*T*_G_ and Δ*H*_G_), freezing (*T*_F_ and Δ*H*_F_) and thawing (*T*_T_ and Δ*H*_T_). Similarly, the process type significantly affected those parameters, except for enthalpy during thawing. In general, the incorporation of yacon flour resulted in lower starch gelatinization enthalpies and, in some cases, higher gelatinization peaks compared to wheat dough. Both gelatinization enthalpy reduction and gelatinization delay occur due to inulin and FOS interactions with water molecules through hydrogen bonds, which reduces water available for starch granule swelling and delays the transition from a crystalline to an amorphous state during gelatinization.^32^ This gelatinization delay is consistent with the results obtained during the dough consistency test (Table 2), since the incorporation of yacon flour increased *t*_3_ from 26.08 min for W to 28.12 min for Y10, while *T*_G_ increased from 74.5 °C for W to 80.4 °C for Y5. These changes indicate that higher temperatures and longer heating times were required to reach starch gelatinization in doughs containing yacon.

**Table 4:** Gelatinization temperature peak (*T*_G_) and enthalpy by starch mass (Δ*H*_G_); freezing temperature peak (*T*_F_) and enthalpy standardized in dry basis (Δ*H*_F_); thawing temperature peak (*T*_T_) and enthalpy standardized in dry basis (Δ*H*_T_); mass of freezable water (*m*_FW_) of the dough produced with different amounts of yacon flour, according to the process types

Process type	Sample	*T*_G_ (°C)	Δ*H*_G_ (J/g starch)	*T*_F_ (°C)	Δ*H*_F_ (J/g d.b.)	*T*_T_ (°C)	Δ*H*_T_ (J/g d.b.)	*m*_FW_ (g)
Direct	W	74.5 ± 0.1^bA^	0.68 ± 0.08^aA^	—	—	—	—	—
	Y5	80.4 ± 0.5^aA^	0.39 ± 0.10^bB^	—	—	—	—	—
	Y10	73.6 ± 1.9^bC^	0.55 ± 0.05^abA^	—	—	—	—	—
Freezing the dough	WF	74.0 ± 0.1^cA^	0.73 ± 0.01^aA^	−8.7 ± 0.2^aA^	78.86 ± 1.90^aA^	−0.8 ± 0.1^aA^	85.91 ± 1.33^aA^	1.89 ± 0.13^aA^
	Y5F	79.4 ± 1.2^aA^	0.50 ± 0.16^bAB^	−9.9 ± 0.2^aA^	68.18 ± 12.81^aA^	−2.0 ± 0.4^bA^	72.55 ± 10.72^aA^	1.67 ± 0.31^aA^
	Y10F	76.1 ± 1.5^bB^	0.73 ± 0.01^aA^	−14.4 ± 0.6^bA^	49.38 ± 1.27^bA^	−4.7 ± 0.1^cA^	48.62 ± 0.27^bA^	1.10 ± 0.00^bA^
Part‐baking and freezing	WFP	73.4 ± 0.6^cA^	0.84 ± 0.14^aA^	−19.1 ± 0.2^aB^	67.88 ± 11.77^aA^	−2.7 ± 0.3^aB^	81.63 ± 12.57^aA^	1.77 ± 0.15^aA^
	Y5FP	79.8 ± 0.6^bA^	0.68 ± 0.15^abA^	−20.9 ± 3.7^aB^	47.02 ± 13.98^bB^	−4.0 ± 0.5^bB^	62.11 ± 8.66^bA^	1.39 ± 0.11^bA^
	Y10FP	82.1 ± 0.5^aA^	0.55 ± 0.11^bA^	−17.6 ± 1.2^aB^	53.33 ± 8.23^abA^	−4.3 ± 0.3^bA^	62.35 ± 6.69^bA^	1.40 ± 0.10^bA^
*P*‐Value, process type		**0.000**	**0.000**	**0.000**	**0.010**	**0.000**	0.904	0.525
*P*‐Value, yacon flour		**0.000**	**0.000**	**0.014**	**0.000**	**0.000**	**0.000**	**0.000**

*Note*: Means with the same lowercase letters are not significantly different in relation to yacon flour content (*p* > 0.05). Means with the same capital letters are not significantly different in relation to process type (*p* > 0.05). The values in bold correspond to those for which a significant difference was found (*p* ≤ 0.05).

Abbreviations: wheat (W), containing 50 g/kg yacon flour (Y5), containing 100 g/kg yacon flour (Y10), freezing the dough process (F), freezing part‐baked process (FP), dry basis (d.b.).

Although freezing and thawing enthalpies were standardized on a dry basis, doughs containing yacon flour generally exhibited lower freezing enthalpy values and reduced mass of freezable water (*m*_FW_) compared to wheat doughs. These results indicate a lower amount of freezable water due to the water‐binding capacity of fructans, in agreement with previous findings for wheat doughs enriched with fiber.^33^ Thus, the reduction in available water reinforces the water‐holding potential of yacon flour and may contribute to improved moisture retention and freshness of the final bread.^7^ For instance, the Δ*H*_F_ and *m*_FW_ decreased by 37.4% and 41.8% from WF to Y10F, suggesting lower ice formation in doughs containing yacon, which may reduce the structural damage during frozen storage.^14^ The fermentation tests revealed a similar proofing performance of Y5F compared to Y5, a behavior possibly associated with this damage reduction, which contributes to maintaining the dough's quality before freezing. This suggests that the low yacon incorporation partially preserved the dough's functionality during freezing, demonstrating the cryoprotective potential of fructans.^9^ However, despite this potential, a higher incorporation of yacon may also affect the gluten network, impairing gas retention and resulting in breads with lower dough expansion, increased hardness and reduced springiness and cohesiveness.^12^, ^13^, ^14^ Despite the higher variability observed for thawing enthalpy in Y5F compared to WF, no statistically significant differences were identified between means according to the Tukey test (*p* > 0.05), likely due to the experimental variability. Since Δ*H*_T_ was not significantly influenced by process type, but varied with yacon level, the data suggest that partial replacement of wheat flour by yacon primarily affects the availability and state of freezable water, rather than any freezing or baking process.

### Bread characterization

Table 5 describes the characteristics of breads obtained using different processes and recipes. Cross‐sections of the bread slices are shown in Fig. 3. The moisture content was higher in wheat breads, likely due to the higher water levels added to the formulation according to the optimal water absorption determined by the consistency test. In addition to formulation effects, statistically significant differences in moisture content were observed among processing types (*p* < 0.05), suggesting that freezing or partial baking may influence water distribution and retention in the bread matrix. Notably, for formulations containing 50 g/kg of yacon flour, the part‐baking followed by freezing was associated with higher moisture retention compared to freezing the dough directly, which may contribute to a fresher texture perception in the final product.

**Table 5:** Impact of wheat flour replacement with yacon flour (YF) on the bread features, according to process type (PT)

PT	Direct			Freezing the dough			Part‐baking and freezing			*P*‐Value	
Samples	W	Y5	Y10	WF	Y5F	Y10F	WFP	Y5FP	Y10FP	PT	YF
Moisture (g/kg)	349.2 ± 11.0^aA^	336.4 ± 4.5^aAB^	317.9 ± 5.4^bA^	349.6 ± 5.3^aA^	330.7 ± 10.2^bB^	318.1 ± 13.1^bA^	355.9 ± 6.6^aA^	351.4 ± 9.1^aA^	329.8 ± 6.5^bA^	**0.000**	**0.000**
L*	72.6 ± 0.8^aA^	68.1 ± 0.7^bA^	66.5 ± 0.5^cA^	72.4 ± 0.9^aA^	67.7 ± 0.7^bA^	65.9 ± 0.6^cA^	73.2 ± 1.5^aA^	67.7 ± 0.8^bA^	66.3 ± 0.5^cA^	0.061	**0.000**
a*	−1.3 ± 0.1^cA^	−0.6 ± 0.1^bA^	−0.1 ± 0.1^aA^	−1.2 ± 0.2^cA^	−0.6 ± 0.1^bA^	−0.1 ± 0.1^aA^	−1.3 ± 0.1^cA^	−0.6 ± 0.1^bA^	−0.1 ± 0.1^aA^	0.251	**0.000**
b*	18.1 ± 0.6^abA^	18.0 ± 0.4^bA^	18.7 ± 0.3^aA^	18.9 ± 0.5^aA^	17.6 ± 0.4^bA^	18.6 ± 0.4^aA^	18.7 ± 0.7^aA^	17.6 ± 0.3^bA^	18.8 ± 0.5^aA^	0.605	**0.000**
Δ*E*	0.0 ± 0.0^cB^	4.61 ± 0.69^bA^	6.28 ± 0.47^aA^	1.11 ± 0.58^cA^	4.99 ± 0.70^bA^	6.83 ± 0.62^aA^	1.66 ± 0.71^cA^	4.98 ± 0.80^bA^	6.46 ± 0.49^aA^	**0.000**	**0.000**
Crust thickness (mm)	1.23 ± 0.20^aAB^	1.37 ± 0.18^aA^	1.42 ± 0.14^aA^	1.30 ± 0.09^bA^	1.48 ± 0.18^aA^	1.50 ± 0.06^aA^	1.00 ± 0.11^bB^	1.28 ± 0.10^aA^	1.39 ± 0.08^aA^	**0.000**	**0.000**
Slice 2D area (cm^2^)	15.02 ± 0.26^bA^	15.95 ± 0.62^aA^	15.50 ± 0.43^abA^	14.82 ± 0.31^aA^	14.78 ± 0.40^aB^	14.98 ± 0.44^aA^	14.73 ± 0.46^aA^	15.28 ± 0.61^aAB^	14.96 ± 0.47^aA^	**0.000**	**0.012**
Hardness (N)	3.07 ± 0.31^bB^	3.62 ± 0.47^bB^	5.05 ± 0.34^aB^	3.78 ± 0.25^bA^	4.22 ± 0.60^bA^	5.23 ± 0.37^aAB^	3.18 ± 0.20^cB^	3.85 ± 0.34^bAB^	5.77 ± 0.90^aA^	**0.000**	**0.000**
Springiness (mm)	6.04 ± 0.40^aA^	5.71 ± 0.44^abA^	5.48 ± 0.49^bA^	5.74 ± 0.23^aA^	5.70 ± 0.57^aA^	5.47 ± 0.38^aA^	5.78 ± 0.30^aA^	5.57 ± 0.57^aA^	4.97 ± 0.23^bB^	**0.006**	**0.000**
Cohesiveness	0.87 ± 0.02^abA^	0.88 ± 0.01^aA^	0.86 ± 0.01^bA^	0.88 ± 0.01^aA^	0.87 ± 0.01^aA^	0.86 ± 0.02^aA^	0.85 ± 0.00^aB^	0.85 ± 0.01^aB^	0.82 ± 0.02^bB^	**0.000**	**0.000**
Chewiness (mJ)	16.15 ± 1.34^bB^	18.12 ± 2.26^bB^	23.77 ± 2.45^aA^	19.12 ± 1.50^bA^	20.91 ± 3.05^bA^	24.79 ± 1.88^aA^	15.61 ± 1.11^bB^	18.27 ± 1.85^bB^	23.36 ± 2.93^aA^	**0.000**	**0.000**

*Note*: Means with the same lowercase letters are not significantly different in relation to yacon flour content (*p* > 0.05). Means with the same capital letters are not significantly different in relation to process type (*p* > 0.05). The values in bold correspond to those for which a significant difference was found (*p* ≤ 0.05).

Abbreviations: water retained (WR), total color difference (Δ*E*), two‐dimensional (2D), wheat (W), bread containing 50 g/kg yacon flour (Y5), bread containing 100 g/kg yacon flour (Y10), freezing the dough process (F), freezing part‐baked process (FP).

**Figure 3:**
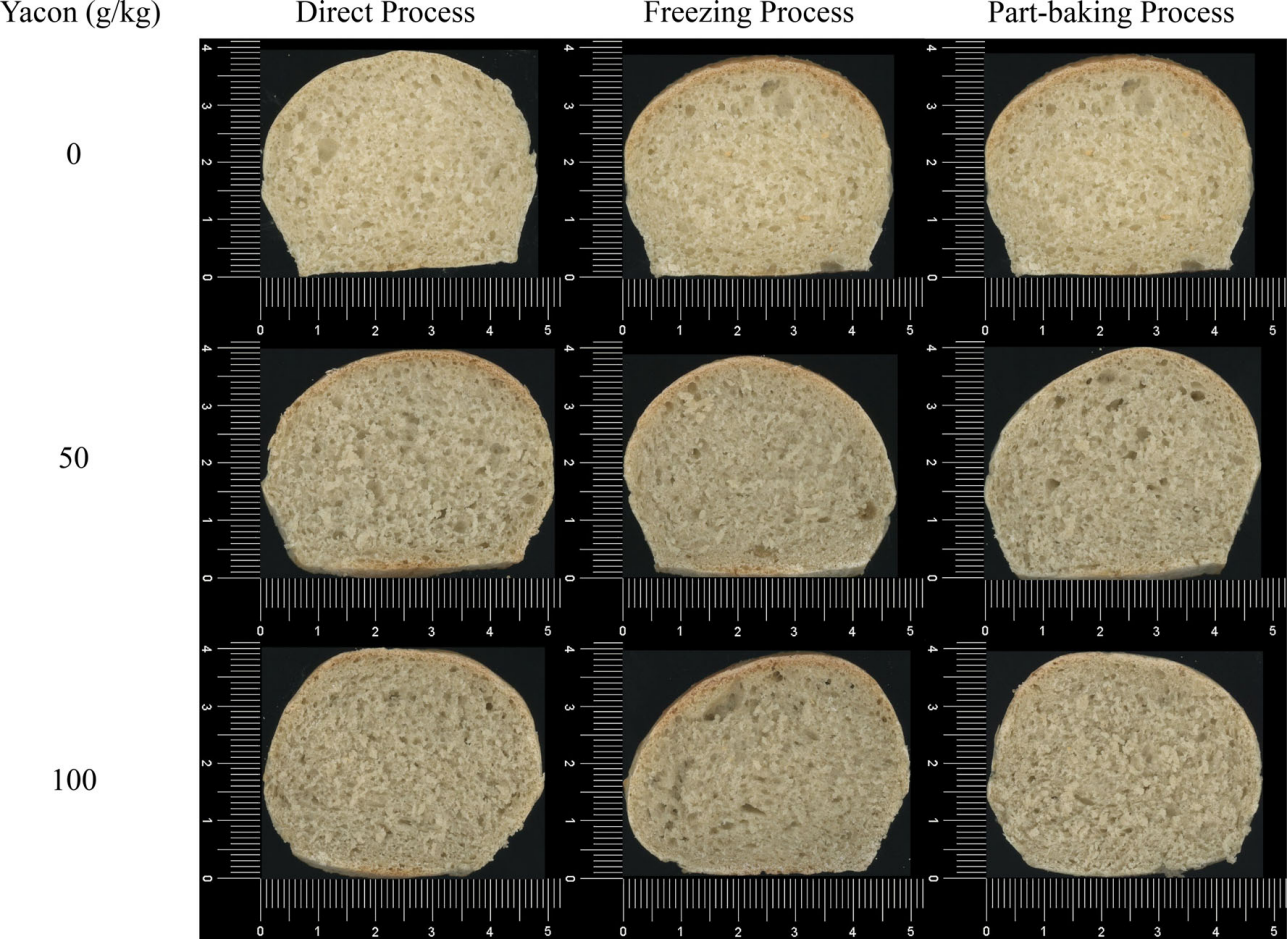
Images of bread slices according to the yacon content and process type.

Both yacon flour content and process type significantly influenced bread texture (*P* < 0.05). The incorporation of yacon flour in formulations generally increased the hardness of bread, presumably due to its higher fiber content.^31^ Processes comprising freezing resulted in a harder crumb, with the hardness increasing as the yacon content increased. The same behavior was previously reported for wheat doughs enriched with fibers produced through the part‐baking process.^30^

Besides, the water retained by the fructans may have influenced the hardness of the bread, since this water was not entirely available to interact with the wheat starch, reducing the mobility of this compound and hardening the bread. Furthermore, these interactions between fructans and water may also reduce the availability of water for gluten hydration by competing for hydrogen bonding sites, thus limiting proper gluten plasticization and potentially contributing to increase crumb hardness.^29^ Consequently, the chewiness found for breads containing 100 g/kg of yacon was greater, suggesting that these formulations are more difficult to chew, mainly due to harder crumbs. Therefore, excessive yacon substitution might negatively affect the textural profile, which has previously been associated with lower consumer acceptance in bread containing yacon flour.^34^

The crumb color was significantly influenced by the yacon content, but no significant effect of the process type was observed. The incorporation of yacon resulted in darker bread, in proportion to the yacon content, as indicated by the decrease in the L* values compared to the control formulation. This finding can be attributed to the higher sugar content in the yacon‐enriched doughs, which promotes caramelization and Maillard reactions, resulting in a darker crumb.^5^, ^7^, ^11^ The image analysis of bread crumb slices (Fig. 3) revealed differences in slice 2D area and crust thickness among formulations and process types. In particular, part‐baked and frozen breads made only with wheat flour displayed lower crust thickness than breads containing yacon flour.

To have a complete picture of the yacon impact on breads made with different levels of yacon and obtained from different breadmaking processes, a PCA was built (Fig. 4). Two main components explaining 83.62% of the variation in bread characteristics were identified. The principal component one (PC1) described 65.75% of the variation and was mainly related to bread moisture, crust thickness, texture properties, and color attributes (L* and a*). Moreover, the principal component two (PC2) represented 17.87% of the variation and was mostly related to slice area, crust thickness, color parameter b*, hardness and springiness. Furthermore, samples were discriminated into three clusters according to yacon content, which were distributed along the abscissa axis (PC1). Hence, the yacon content has a major effect on the bread's textural properties and crumb color. Likewise, within these clusters, the samples were dispersed along the ordinate axis (PC2) based on process type, implying that freezing or part‐baking followed by freezing significantly affected slice area and certain textural properties (such as hardness and springiness). Such process‐driven differences reflect the potential influence of ice crystal formation (and subsequent thawing) on the gluten network integrity and final bread geometry, as also suggested by the fermentation and enthalpy data in the frozen dough formulations. The PCA confirms that yacon content primarily governs color and texture changes, while the process type further modulates structural and dimensional properties.

**Figure 4:**
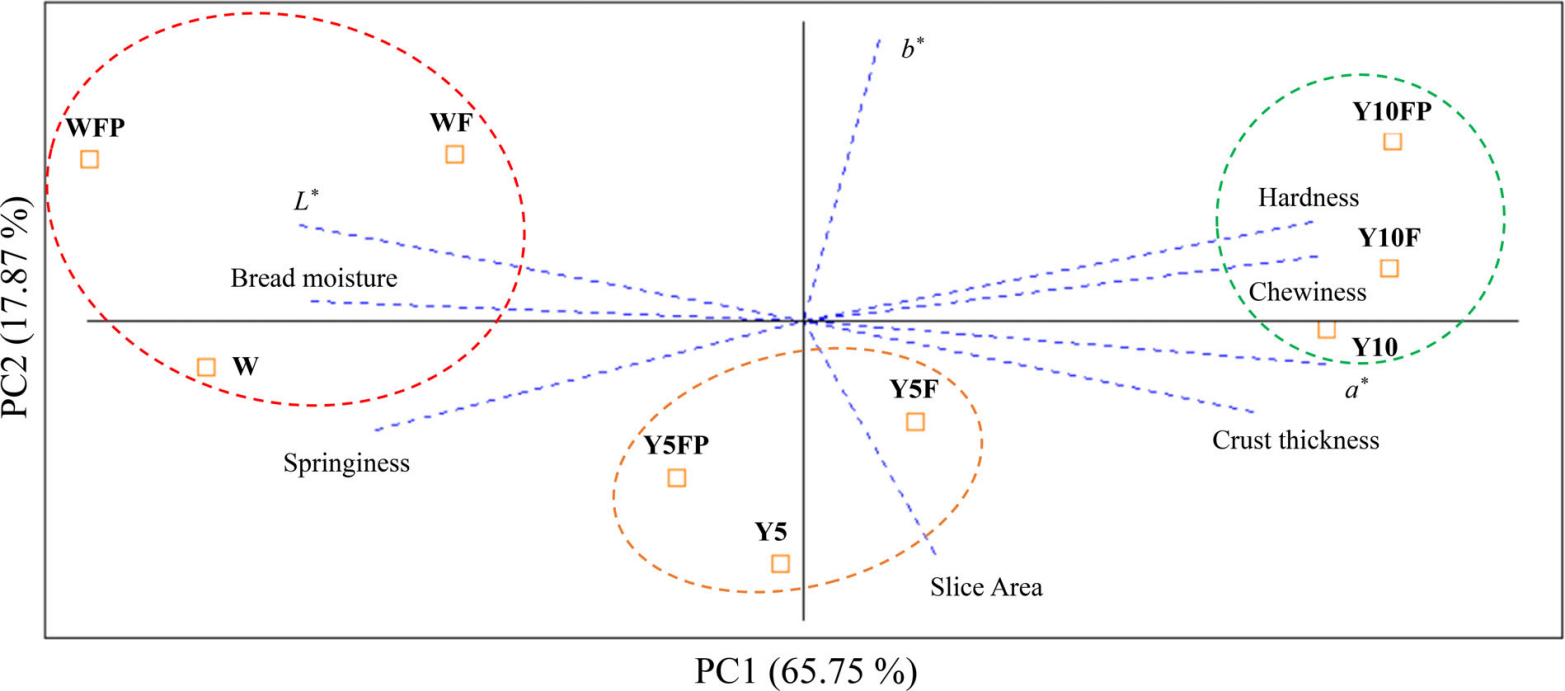
Principal component analysis (PCA) of bread characteristics. The bread samples are represented by squares and identified by their respective codes. The ellipses formed by dashed lines distinguish the groups formed according to yacon content. Abbreviations: wheat (W), containing 50 g/kg yacon flour (Y5), containing 100 g/kg yacon flour (Y10), freezing the dough process (F), freezing part‐baked process (FP).

## CONCLUSIONS

The cryoprotectant potential of yacon flour was evaluated in different breadmaking processes, including a freezing stage at the dough or bread level. One technological potential benefit of yacon's fructan content was observed in the fermentation test for the direct process, since, even with a lower gluten content, the enriched doughs exhibited similar dough volume increase, probably due to the yeast activity enhancement provided by the fructose contained in these carbohydrates. Furthermore, the potential cryoprotective activity of this flour was identified during the fermentation test comprising freezing process, since the sample Y5F presented the same fermentation performance as Y5, while the same did not occur for other formulations. This suggests that the cryoprotective activity and yeast activity enhancement were not enough to overcome the weakness of the gluten network due to a substitution of wheat flour by a yacon flour content of 100 g/kg, which probably hinders the gas retention capacity, and consequently limits the dough expansion. This may occur due to the competitiveness for water bonding after replacement of wheat flour by yacon flour, which affects the capacity of wheat starch and gluten to interact with water, directly impacting bread hardness and the constitution of the gluten matrix. In alignment with this, the Mixolab and DSC results suggested that yacon flour weakens the gluten network and hinders starch gelatinization. In addition, the thermal properties were impacted by the lower freezable water content in dough containing yacon. Thus, the replacement of wheat flour with yacon flour is limited due to the strength of the gluten network and the final characteristics of the bread, mainly color and texture. Therefore, despite the possible benefits of this tuber in breadmaking, its use should be done carefully to avoid negative effects. Considering this, the formulations containing 50 g/kg of yacon content presented good results for most of the tests carried out when compared to the control samples. Hence, this formulation could be applied in the production of bread containing yacon to provide potential nutritional and technological enrichment to the bread without losing its traditional characteristics, both in the direct process and in those involving freezing.

## AUTHOR CONTRIBUTIONS

Gabriel Aparecido Atanazio da Silva: formal analysis, investigation, writing – original draft, review and editing, visualization. Raquel Garzon: conceptualization, methodology, resources, writing – review and editing, supervision. Giulianna Petean Torrano: formal analysis, review and editing. Carmen Cecilia Tadini: conceptualization, methodology, resources, writing – review and editing, supervision, funding acquisition. Cristina M. Rosell: conceptualization, resources, writing – review and editing, supervision.

## CONFLICT OF INTEREST

The authors declare that they have no known competing financial interests or personal relationships that could have appeared to influence the work reported in this article.

## Data Availability

The data that support the findings of this study are available from the corresponding author upon reasonable request.
